# Professional Brushing Following Shortened Outreach Periodontal Therapy in Nursing Home Residents: A Prospective Extension Study With Historical Comparisons

**DOI:** 10.1111/scd.70270

**Published:** 2026-09-30

**Authors:** Anna Greta Barbe, Franka Lefering, Martin Hellmich, Max von Kohout, Sonja H. M. Derman, Dirk Bleiel

**Affiliations:** ^1^ Faculty of Medicine and University Hospital Cologne, Polyclinic for Operative Dentistry and Periodontology University of Cologne Cologne Germany; ^2^ Faculty of Medicine and University Hospital Cologne, Institute of Medical Statistics and Computational Biology (IMSB) University of Cologne Köln Germany; ^3^ Department of Medical Statistics University Medical Center Göttingen Georg‐August‐University Göttingen Germany; ^4^ Joint Dental Practice Dres. Pia und Dirk Bleiel Rheinbreitbach Germany

**Keywords:** brushing, dental plaque, gingival bleeding, nursing homes, periodontitis

## Abstract

**Aims:**

To substantiate the effectiveness of shortened outreach periodontal therapy in nursing home residents with periodontitis, we evaluated additional professional brushing every two weeks in a prospective extension study using historical comparison groups from a previously published randomized controlled trial.

**Methods and Results:**

Participants receiving shortened outreach periodontal therapy with additional professional brushing every two weeks (intervention with brushing, IWB) were prospectively enrolled and compared with historical participants from a previously published randomized trial, including an intervention‐only group (IO) and untreated controls. Primary outcome: bleeding on probing (BOP); secondary outcomes: plaque index (PI), probing pocket depth (PPD). Assessments were made at baseline and three months. Fifty‐four participants (18 per group) were included. At three months, only IWB reduced BOP compared with baseline (ΔBOP −20 ± 20 percentage points; *p* = 0.002), with greater reductions than controls (*p* < 0.001) but not IO (*p* = 0.203). Both PI and PPD decreased with IWB (*p* = 0.005 and *p* = 0.001 vs. baseline, respectively) and IO (*p* = 0.014 and *p* = 0.005), but not in controls. The reduction in PPD (but not PI) was greater with IWB and IO than in controls (*p* < 0.001).

**Conclusion:**

Additional professional brushing every two weeks provided clinical benefits beyond shortened outreach periodontal therapy, particularly regarding periodontal inflammation, but did not fully compensate for inadequate daily oral hygiene.

**Trial Registration**: German Clinical Trials Register (DRKS), registration number DRKS00029392

## 1 Introduction

While improvements in life‐long oral health have enabled more older adults to keep their natural teeth [1, 2, 3, 4], adequate daily oral hygiene can be challenging in long‐term care facilities where access to dental services is often limited and little outreach care is available [2]. Accordingly, gingivitis and moderate‐to‐severe periodontitis (characterized by substantial attachment loss) are common in nursing home residents [5]. In addition to the associated pain and impaired oral health, this vulnerable population is consequently at greater risk of systemic health complications, including malnutrition, respiratory infections such as aspiration pneumonia, cardiovascular and neurodegenerative diseases, and an overall increased systemic inflammatory burden [6, 7, 8, 9, 10, 11].

Despite the high demand for dental care in nursing homes, there is limited knowledge about the clinical efficacy of periodontal therapies provided in outreach settings. While studies have demonstrated that periodontal interventions are effective in younger and generally healthy individuals [12, 13], there is a paucity of research regarding their impact in older, functionally impaired populations with care needs. One of the key factors influencing the success of such therapies might be the ability of patients to maintain adequate daily oral hygiene—an aspect that is often compromised among nursing home residents due to physical and cognitive limitations, but is currently inadequately redressed by support from nursing, medical, and dental professionals [14, 15, 16]. Structured oral hygiene measures, such as professional brushing performed by dental specialists, may be necessary to achieve sustained improvements in oral health within this vulnerable group [17, 18, 19, 20, 21], thereby helping to mitigate the risks associated with periodontitis (e.g., pneumonia [11]).

In a recent randomized controlled trial (RCT) [22], the clinical effectiveness of shortened outreach periodontal therapy (involving subgingival instrumentation) over a three‐month period was compared to no periodontal treatment in nursing home residents diagnosed with periodontitis—a particularly vulnerable population characterized by limited functional capacity, dependency on care, and occasional non‐cooperation. It was found that shortened outreach periodontal therapy reduced gingival inflammation and probing depths; however, the reduction in plaque levels was not significantly greater after periodontal therapy compared with no intervention. The authors concluded that the modest changes in clinical parameters were most likely due to suboptimal regular oral hygiene within this population, and that the oral hygiene intervention was insufficient to maintain the therapeutic benefits over time (inferred from the lack of significant improvements in bleeding on probing). Shortened outreach periodontal therapy refers to a specific periodontal treatment pathway established within the German statutory health insurance system (§22a SGB V) for individuals requiring long‐term care or with disabilities. Compared with the standard systematic periodontal treatment pathway, this approach shortens the administrative process for individuals in long term care or with disabilities to access periodontal care by reducing the administrative burden and diagnostic procedures needed prior to subgingival instrumentation in an outreach setting.

Accordingly, we conducted a prospective extension study based on our previously published randomized controlled trial. Participants receiving shortened outreach periodontal therapy combined with structured professional brushing every two weeks (IWB) were prospectively enrolled and compared with participants from the original trial (IO and controls) [22]. Participants from the original no‐intervention control group were enrolled who, for ethical reasons, had deferred their periodontal treatment until the end of the initial three‐month study period. This allowed us to compare results from the new IWB group against those in the original trial (intervention only (IO) and no intervention (controls)) [22]. We aimed to determine whether the addition of professional brushing every two weeks would yield significant benefits in sustaining or enhancing periodontal treatment outcomes in this population. Our primary endpoint was periodontal inflammation, assessed by bleeding on probing (BOP), with measures of plaque accumulation and probing pocket depths as secondary outcomes. Our objective was to inform the development of more effective outreach oral health strategies for elderly individuals in long‐term care settings. This is the first study to evaluate the combined effect of structured professional oral hygiene provided every two weeks and periodontal therapy in an outreach context.

## 2 Materials and Methods

The study received ethical approval from the local ethics review board and was registered prior to patient enrollment. All procedures complied with the ethical standards of the institutional research committee, the 1964 Helsinki Declaration, and subsequent amendments or comparable ethical standards. Informed consent was obtained from all participants or their legal guardians. The data that support the findings of this study are available from the corresponding author upon reasonable request.

The present investigation was designed as a prospective extension study using historical comparison groups derived from a previously published randomized controlled trial. As an extension of the original study, the methods for the current study are as previously described in Barbe et al. [22]. Briefly, shortened outreach periodontal therapy refers to the periodontal treatment pathway according to §22a SGB V in Germany and involves shortened administrative and diagnostic requirements prior to subgingival instrumentation, thereby facilitating access to periodontal therapy in an outreach setting.

### 2.1 Study Population

All participants (>65 years old) were nursing home residents, had periodontitis requiring outreach dental and periodontal care, and were covered by standard statutory health insurance. All participants received dental care from the study dentist. Eligibility criteria included no antibiotic therapy in the past six months and a diagnosed need for periodontal treatment based on the Periodontal Screening Index [23]; a probing depth of ≥4 mm indicated the necessity for periodontal therapy [24]. Exclusion criteria included terminal illnesses suggesting imminent death or cases where statutory health insurance did not cover periodontal treatment.

### 2.2 Sample Size

The original sample size calculation was performed for the original study design [22], which determined that 18 participants per group would be required to account for an anticipated dropout rate of 5% per group. No additional sample size calculation was performed for the new intervention group.

In the original RCT [20], eligible participants were randomized into two groups (IO or controls), using a randomization list created prior to the start of the study; one patient dropped out of the control group. At the follow‐up assessment, 12 out of 17 remaining participants in the control group dropped out due to unwillingness to continue participation, unwillingness to receive further periodontal therapy, illness, or death.

The five remaining participants in the original control group were enrolled in the current study. To compensate for the dropouts from the original group, 13 new participants were recruited from our waiting list, bringing the sample size back to 18. Consequently, participants in the IWB group were not randomized. The IO and control groups therefore served as historical comparison groups for the present extension study.

### 2.3 Study Interventions

**Study appointments**. Clinical assessments were performed at three time points in both intervention groups. At baseline, BOP, PI, and PPD were assessed before anti‐infective therapy (AIT), comprising subgingival instrumentation, was performed. At the 7‐day study visit, PI was assessed without additional professional cleaning or subgingival instrumentation. At the 3‐month follow‐up, BOP, PI, and PPD were reassessed before any further periodontal treatment. Under routine §22a SGB V care, supportive periodontal treatment, including periodontal reassessment, indicated subgingival reinstrumentation, and supragingival cleaning, is scheduled three to six months after AIT; however, in the present study, all 3‐month outcome assessments were completed before any such further treatment.

**IO group**. In the original study, participants first received individualized oral hygiene instruction prior to AIT. An individual oral hygiene plan was established for each participant, and recommended oral hygiene procedures were demonstrated using the participant's existing personal oral hygiene products. No standardized or additional oral hygiene products or aids were provided as part of the IO intervention. Nursing home staff also received annual oral care training from the cooperating dentist. Participants subsequently received anti‐infective periodontal therapy (AIT), comprising subgingival instrumentation, in the nursing home. All procedures were performed using a mobile dental unit. Subgingival instrumentation was carried out using hand instruments in combination with machine‐assisted scaling. Tooth surfaces were polished, pockets were irrigated, and interdental spaces were cleaned.

**IWB group**. In the current study, all participants in the IWB group received the same periodontal intervention (anti‐infective therapy, AIT) as the original IO group, followed by an additional professional brushing regimen every two weeks. Unlike participants in the IO group, participants in the IWB group were provided with standardized oral hygiene equipment as part of the intervention, including an electric toothbrush, interdental brushes, and, where applicable, a prosthetic brush. These products remained with the participants for continued use with caregiver assistance. Toothbrushing was performed using the Stillman sweeping technique and the KAI technique (covering occlusal, outer, and inner surfaces) with the Oral‐B Vitality PRO Lilac electric toothbrush and Blend‐a‐med Complete toothpaste (fluoride concentration: 1450 ppm; approximately 1 g per application). Interproximal spaces were cleaned using chlorhexidine (CHX) gel (2%) and TePe interdental brushes in yellow and black, selected according to individual patient needs. The appropriate brush size was determined based on a standardized assessment chart. CHX gel (approximately 2–4 g in total) was applied directly to the interdental brushes and introduced into the interproximal spaces. For participants with dental prostheses, additional cleaning was performed using a prosthetic toothbrush (Blend‐a‐med). All procedures were carried out by dental professionals, and the intervention was repeated every two weeks, totaling six sessions over a three‐month period. All procedures were performed under routine nursing home conditions. Depending on the residents’ mobility and functional status, professional brushing was carried out in wheelchairs, standard armchairs, bathroom facilities, or, in a small number of severely impaired residents, at the bedside. Illumination was provided by standard room lighting and, when required, a simple headlamp. No magnification devices or dental treatment chairs were used. This pragmatic approach was chosen to reflect the realities of outreach oral healthcare in nursing home settings.

**Historic controls**. Participants in the original control group were examined at baseline and follow‐up at three months, but did not receive any dental treatment.

### 2.4 Endpoints

Personal health measures (care dependency level, cognitive status, prescribed medications, systemic diseases) were obtained from the nursing home medical files. Care dependency was classified according to the German long‐term care system using five care grades, ranging from 1 (slight impairment of independence) to 5 (most severe impairment of independence with special requirements for nursing care). The primary endpoint was BOP. Secondary endpoints included the plaque index (PI), mean and maximal probing pocket depth (PPD), and assessment (by the study dentist) of cooperation during instrumentation in the AIT sessions and the number of necessary AIT appointments [25]. BOP, PI, and PPD were assessed full‐mouth at two sites per tooth (mesioapproximal and distoapproximal), in accordance with the shortened outreach periodontal therapy pathway under §22a SGB V. Thus, conventional six‐site periodontal charting was not performed. Mean and maximum PPD were calculated from these two‐point full‐mouth measurements. The time required for the AIT procedure (min) was also recorded. BOP, PI, and PPD were measured at the beginning of each examination session [26, 27, 28].

### 2.5 Statistical Analysis

*P*‐values < 0.05 were considered statistically significant. Statistical analyses were performed using IBM SPSS Statistics for Windows, Version 28.0 (IBM Corp., Armonk, NY, USA) [29], and R (R Core Team 2023) [30]. Descriptive statistics (mean, standard deviation, and range) were calculated for BOP, PI, and PPD for each study group and examination time point. Inter‐individual differences between groups were assessed using the Mann–Whitney U‐test, and intra‐individual changes (Δ) over time were evaluated using the Wilcoxon signed‐rank test.

Generalized linear mixed models with repeated measures and a compound symmetry correlation structure were used to analyze the study endpoints. A model‐building strategy based on AIC, BIC, and R^2^ was applied to avoid overfitting and determine the most parsimonious model for each endpoint. The initial model included the interaction between intervention and time point as fixed effects and a random intercept to account for participant‐level variation. Subsequent models were built by stepwise addition of variables of interest, considering their roles as covariates and potential interactions. For the tooth‐type‐specific analysis, tooth group (incisors, canines, premolars, and molars) and its interaction with intervention and time point were included to assess whether intervention effects differed according to tooth type. Model assumptions, including normality of residuals (overall and for random effects), homogeneity of variance, and linearity, were assessed visually and were considered adequately met.

Due to model complexity, only coefficients for the interaction between intervention and time point, representing the overall intervention effect, are presented; 95% confidence intervals and p‐values were computed using a Wald t‐distribution approximation. To facilitate interpretation, estimated marginal means with 95% confidence intervals were examined for the three study groups (control, IO, and IWB) across the two time points (baseline and 3‐month follow‐up) and tooth groups, while controlling for the other model parameters. For multiple comparisons derived from the models, the Benjamini–Hochberg procedure was applied to control the false discovery rate while maintaining statistical power.

## 3 Results

Results were available for 54 participants overall, including 18 prospectively enrolled participants in the IWB group and 36 participants from the previously published trial who served as historical comparison groups [22]. (Figure 1). Baseline characteristics are presented in Table 1. The mean age of participants was 85.3±5.9 years, 77.8% were female, and the mean number of remaining teeth was 16.3±7.5. Overall, 63.0% of participants had a diagnosis of dementia and their overall care level was relatively high (3.6±0.9); when analyzing care levels as categorical variables, most participants were classified as care level 3 (42.6%) or 4 (31.5%). The mean duration of AIT was 14.0 ± 5.4 min in the IO group and 14.7 ± 5.0 min in the IWB group. There were no significant differences between groups, apart from the number of active pharmaceutical ingredients, which was significantly lower in the IWB group (8.1 ± 4.2) than in the IO group (11.4 ± 3.4; *p* = 0.013).

**FIGURE 1:**
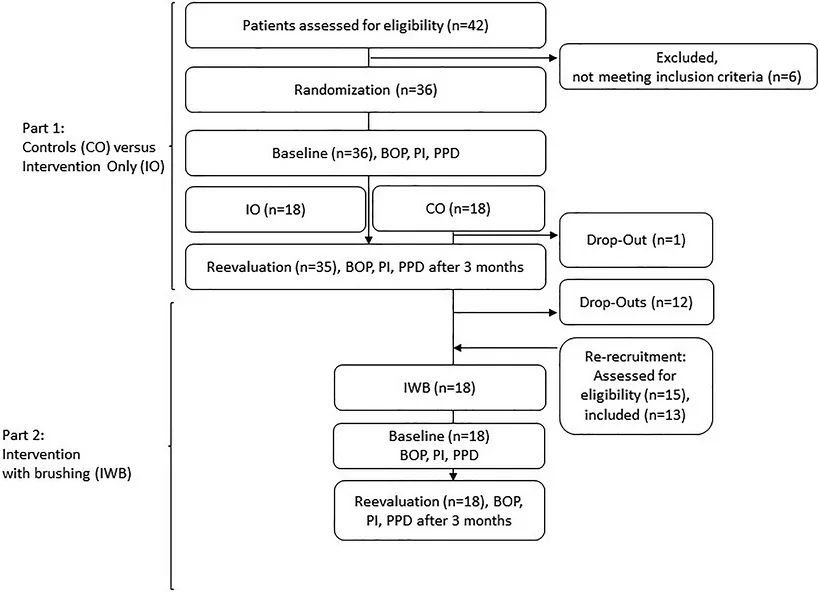
Study flowchart. BOP, bleeding on probing; CO, controls; IO, intervention only; IWB, intervention with brushing; PI, plaque index; PPD, probing pocket depth.

**TABLE 1:** Baseline characteristics for the three different study groups IWB, IO and CO.

	All(n = 54)	IWB(n = 18)	IO(n = 18)	Controls(n = 18)	*p*‐value^a^		
	Mean ± SDMin to max				IWB / IO	IWB / CO	IO / CO
Age (years)	85.3 ± 5.9 69 ‐ 95	86.3 ± 5.3 76 ‐ 95	85.2 ± 5.6 73 ‐ 93	84.5 ± 6.9 69 ‐ 95	0.791	0.424	0.719
APIs	10.1 ± 4.3 3 ‐ 20	8.1 ± 4.2 3 ‐ 18	11.4 ± 3.4 5 ‐ 19	10.8 ± 4.6 6 ‐ 20	**0.013**	0.079	0.628
Number of teeth	16.3 ± 7.5 3 ‐ 30	14.9 ± 7.6 3 ‐ 24	17.4 ± 7.3 6 ‐ 30	16.6 ± 7.9 6 ‐ 28	0.372	0.339	0.767
Duration AIT (minutes)	14.3 ± 5.2 7 ‐ 30	14.7 ± 5.0 7 ‐ 25	14.0 ± 5.4 7 ‐ 30	—	0.791	—	—
Sex, n (%)							
Female	42 (77.8)	14 (77.8)	14 (77.8)	14 (77.8)	1.000	1.000	1.000
Male	12 (22.2)	4 (22.2)	4 (22.2)	4 (22.2)			
Dementia diagnosis, n (%)							
Yes	34 (63.0)	13 (72.2)	11 (61.1)	10 (55.6)	0.480	0.298	0.735^b^
No	20 (37.0)	5 (27.8)	7 (38.9)	8 (44.4)			
Care level, Mean ± SD Min to max	3.6 ± 0.9 2 ‐ 5	3.8 ± 0.9 2 ‐ 5	3.8 ± 0.8 3 ‐ 5	3.2 ± 0.8 2 ‐ 5	0.815	0.064	0.085
Care level n (%)							
2	4 (7.4)	1 (5.6)	0 (0.0)	3 (16.7)	0.706	0.225	0.176
3	23 (42.6)	6 (33.3)	8 (44.4)	9 (50.0)			
4	17 (31.5)	6 (33.3)	6 (33.3)	5 (27.8)			
5	10 (18.5)	5 (27.8)	4 (22.2)	1 (5.6)			

Abbreviations: AIT, anti‐infective therapy; APIs, active pharmaceutical ingredients; IO, intervention only; IWB, intervention with brushing; SD, standard deviation. Bold numbers indicate *p* < 0.05.

^a^ Mann‐Whitney *U*‐test.

^b^ Pearson chi‐square test.

### 3.1 Oral Health

Table 2 shows the clinical measures at baseline and the three‐month follow‐up (one patient in the original control group had dropped out before the three‐month follow‐up, see Figure 1).

**TABLE 2:** Clinical measures at baseline and three months after intervention for the three study groups: IWB, IO and CO.

	IWB	IO	Controls	*p*‐value^a^		
	Mean ± SD Min to max (Number of patients)			IWB/IO	IWB/CO	IO/CO
**BOP (%)**						
Baseline	39 ± 24 **%** 0 – 83 **%** (18)	45 ± 20 **%** 0 – 94 **%** (18)	33 ± 25 **%** 0 – 86 **%** (18)	0.239	0.443	0.083
FU‐1 (3 months)	19 ± 14 **%** 0 – 50 **%** (18)	34 ± 13 **%** 9 – 56 **%** (18)	38 ± 25 **%** 0 – 85 **%** (17)			
*p*‐value^b^	**0.002**	0.058	0.309			
Δ BOP (FU‐1 ‐ BL)	−20 ± 20 pp −60 – 11 pp (18)	−11 ± 24 pp −67 – 28 pp (18)	5 ± 16 pp −28 – 38 pp (17)	0.203	**< 0.001**	**0.038**
**PI**						
Baseline	0.7 ± 0.4 0.1 – 1.4 (18)	1.0 ± 0.4 0.2 – 1.5 (18)	0.8 ± 0.4 0.1 – 1.6 (18)	**0.027**	0.636	0.083
FU‐1 (3 months)	0.5 ± 0.2 0.0 – 0.9 (18)	0.7 ± 0.4 0.2 – 1.2 (17)	0.7 ± 0.4 0.1 – 1.4 (18)			
*p*‐value^b^	**0.005**	**0.014**	0.246			
Δ PI (FU‐1 ‐ BL)	−0.3 ± 0.3 −0.2 – 0.5 (18)	−0.2 ± 0.4 −0.9 – 0.8 (17)	−0.1 ± 0.3 −0.7 – 0.4 (18)	0.708	0.161	0.110
**Mean PPD (mm)**						
Baseline	4.5 ± 0.5 mm 3.8 – 5.6 mm (18)	4.5 ± 0.5 mm 3.8 – 5.6 mm (18)	4.3 ± 0.5 mm 3.6 – 5.9 mm (18)	0.719	0.184	0.062
FU‐1 (3 months)	3.8 ± 0.4 mm 3.1 – 4.5 mm (18)	4.0 ± 0.6 mm 2.8 – 5.2 mm (18)	4.3 ± 0.4 mm 3.6 – 5.1 mm (17)			
*p*‐value^b^	**0.001**	**0.005**	0.136			
Δ mean PPD (FU‐1 ‐ BL)	−0.7 ± 0.4 mm −1.3 – 0.1 mm (18)	−0.5 ± 0.6 mm −2.2 – 0.2 mm (18)	0.1 ± 0.4 mm −0.9 – 0.6 mm (17)	0.214	**< 0.001**	**< 0.001**
**Maximum PPD (mm)**						
Baseline	5.8 ± 0.7 mm 5 – 8 mm (18)	6.0 ± 1.0 mm 4 – 8 mm (18)	5.5 ± 0.6 mm 5 – 7 mm (18)	0.443	0.503	0.153
FU‐1 (3 months)	4.9 ± 0.8 mm 4 – 7 mm (18)	5.4 ± 0.8 mm 4 – 7 mm (18)	5.7 ± 0.8 mm 5 – 7 mm (17)			
*p*‐value^b^	**0.001**	**0.008**	0.317			
Δ max PPD (FU‐1 ‐ BL)	−0.9 ± 0.9 mm −2 – 0 mm (18)	−0.6 ± 0.8 mm −2 – 1 mm (18)	0.2 ± 0.7 mm −1 – 2 mm (17)	0.372	**< 0.001**	**0.009**

Bold numbers indicate *p* < 0.05.

Abbreviations: BL, baseline; BOP, bleeding on probing; CO, controls; FU, follow‐up; PI, plaque index; pp, percentage points; PPD, probing pocket depth; SD, standard deviation.

^a^ Mann‐Whitney U‐test.

^b^ Wilcoxon signed‐rank test.

**BOP**. The mean BOP values were comparable across groups at baseline. After three months, only the IWB group achieved a statistically significant reduction in BOP compared with baseline (IWB: ΔBOP −20 ± 20 percentage points; *p* = 0.002). The reduction was significantly greater in the IWB group than in controls (control: ΔBOP +5 ± 16 percentage points; *p* < 0.001), but did not differ significantly from the IO group (IO: ΔBOP −11 ± 24 percentage points; *p* = 0.203).

**PI**. At baseline, PI was highest in the IO group (1.0 ± 0.4) and was significantly higher than in the IWB group (0.7 ± 0.4; *p* = 0.027). After three months, both IWB (ΔPI ‐0.3±0.3; *p* = 0.005) and IO (ΔPI ‐0.2±0.4; *p* = 0.014) led to a significant reduction in PI compared with baseline. However, intergroup comparison did not reach statistical significance (*p*>0.05 for all comparisons).

**PPD**. The mean PPD was similar between groups at baseline. After three months, PPD decreased significantly compared with baseline in both the IWB group (ΔPPD −0.7 ± 0.4 mm; *p* = 0.001) and the IO group (ΔPPD −0.5 ± 0.6 mm; *p* = 0.005). The reduction was significantly greater in both the IWB and IO groups than in controls (control: ΔPPD 0.1 ± 0.4 mm; *p* < 0.001), whereas there was no significant difference between the IWB and IO groups (*p* = 0.214).

### 3.2 Probing Pocket Depths in Categories

Full details for all PPD categories can be found in Table 3 and Figure 2.

**TABLE 3:** Distribution of PPD categories (healthy, moderate, and deep) at baseline and the 3‐month follow‐up in the three study groups (IWB, IO, and controls).

	IWB	IO	Controls	*p*‐value^a^			
	Mean ± SD Min to max (Number of patients)			IWB/IO	IWB/CO		IO/CO
**Healthy PPD sites (1–3 mm), %**							
Baseline	6.8 ± 9.8 % 0 – 29 % (18)	8.2 ± 10.3 % 0 – 32 % (18)	16.1 ± 18.8 % 0 – 50 % (17)				
FU‐1 (3 months)	36.2 ± 27.5 % 0 – 85 % (18)	25.1 ± 28.4 % 0 – 91 % (18)	9.2 ± 13.1 % 0 – 40 % (17)				
*p*‐value^b^	**0.001**	**0.036**	0.075				
Δ healthy PPD sites (FU‐1 − BL), pp	29.4 ± 28.2 pp −3 – 83 pp (18)	16.8 ± 28.9 pp −16 – 83 pp (18)	−6.9 ± 16.1 pp −48 – 21 pp (17)	0.097	**< 0.001**	**0.019**	
**Moderate PPD sites (4–5 mm), %**							
Baseline	83.3 ± 14.9 % 42 – 100 % (18)	79.4 ± 14.8 % 50 – 95 % (18)	76.2 ± 21.0 % 37 – 100 % (17)				
FU‐1 (3 months)	62.9 ± 26.4 % 15 – 100 % (18)	70.6 ± 26.1 % 9 – 100 % (18)	83.6 ± 11.0 % 60 – 100 % (17)				
*p*‐value^b^	**0.044**	0.296	0.266				
Δ moderate PPD sites (FU‐1 − BL), pp	−20.4 ± 35.7 pp −75 – 44 pp (18)	−8.8 ± 30.0 pp −77 – 50 pp (18)	7.4 ± 21.6 pp −21 – 53 pp (17)	0.214	**0.014**	0.134	
**Deep PPD sites (≥6 mm), %**							
Baseline	9.9 ± 16.4 % 0 – 58 % (18)	12.4 ± 15.2 % 0 – 50 % (18)	7.6 ± 15.4 % 0 – 64 % (17)				
FU‐1 (3 months)	1.0 ± 2.9 % 0 – 11 % (18)	4.4 ± 8.4 % 0 – 33 % (18)	7.2 ± 8.2 % 0 – 23 % (17)				
*p*‐value^b^	**0.005**	**0.016**	0.646				
Δ deep PPD sites (FU‐1 − BL), pp	−9.0 ± 14.3 pp −53 – 0 pp (18)	−8.0 ± 14.5 pp −50 – 4 pp (18)	−0.4 ± 12.5 pp −41 – 19 pp (17)	0.606	**0.027**	0.083	

Abbreviations: AIT, anti‐infective therapy; BL, baseline; CO, controls; FU, follow‐up; pp, percentage points; PPD, probing pocket depth; IO, intervention only; IWB, intervention with brushing; SD, standard deviation. Bold numbers indicate *p* < 0.05.

^a^ Mann‐Whitney U‐test.

^b^ Wilcoxon signed‐rank test.

**FIGURE 2:**
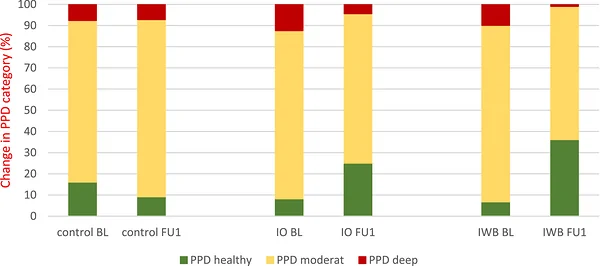
Distribution of PPD categories by study group at baseline and the 3‐month follow‐up. Color coding: green = healthy pockets; yellow = moderate pockets; red = deep pockets. BL, baseline; IO, intervention only; IWB, intervention with brushing; FU, follow‐up; PPD, probing pocket depth.

**Healthy PPD (1–3 mm)**. At baseline, the mean number of sites with a healthy PPD was lowest in the IWB group (2 ± 3 sites) and highest in the control group (6 ± 8 sites). After three months, the mean number of healthy PPD sites increased to 11 ± 11 sites in the IWB group and 6 ± 6 sites in the IO group, whereas it decreased to 3 ± 5 sites in the control group. The percentage of healthy PPD sites increased significantly in the IWB group (Δ healthy PPD sites: +29.4 ± 28.2 percentage points; *p* = 0.001) and the IO group (Δ healthy PPD sites: +16.8 ± 28.9 percentage points; *p* = 0.036). Intergroup comparisons showed that the increase in the percentage of healthy PPD sites was significantly greater in the IWB group (*p* < 0.001) and the IO group (*p* = 0.019) than in controls, whereas the difference between the IWB and IO groups was not statistically significant (*p* = 0.097).

### 3.3 Model Effects

Estimated marginal means with 95% confidence intervals for BOP, PI, and PPD according to tooth group, study group, and time point are shown in Figure 3. Statistically significant intergroup differences between IWB and IO are indicated in Figure 3.

**FIGURE 3:**
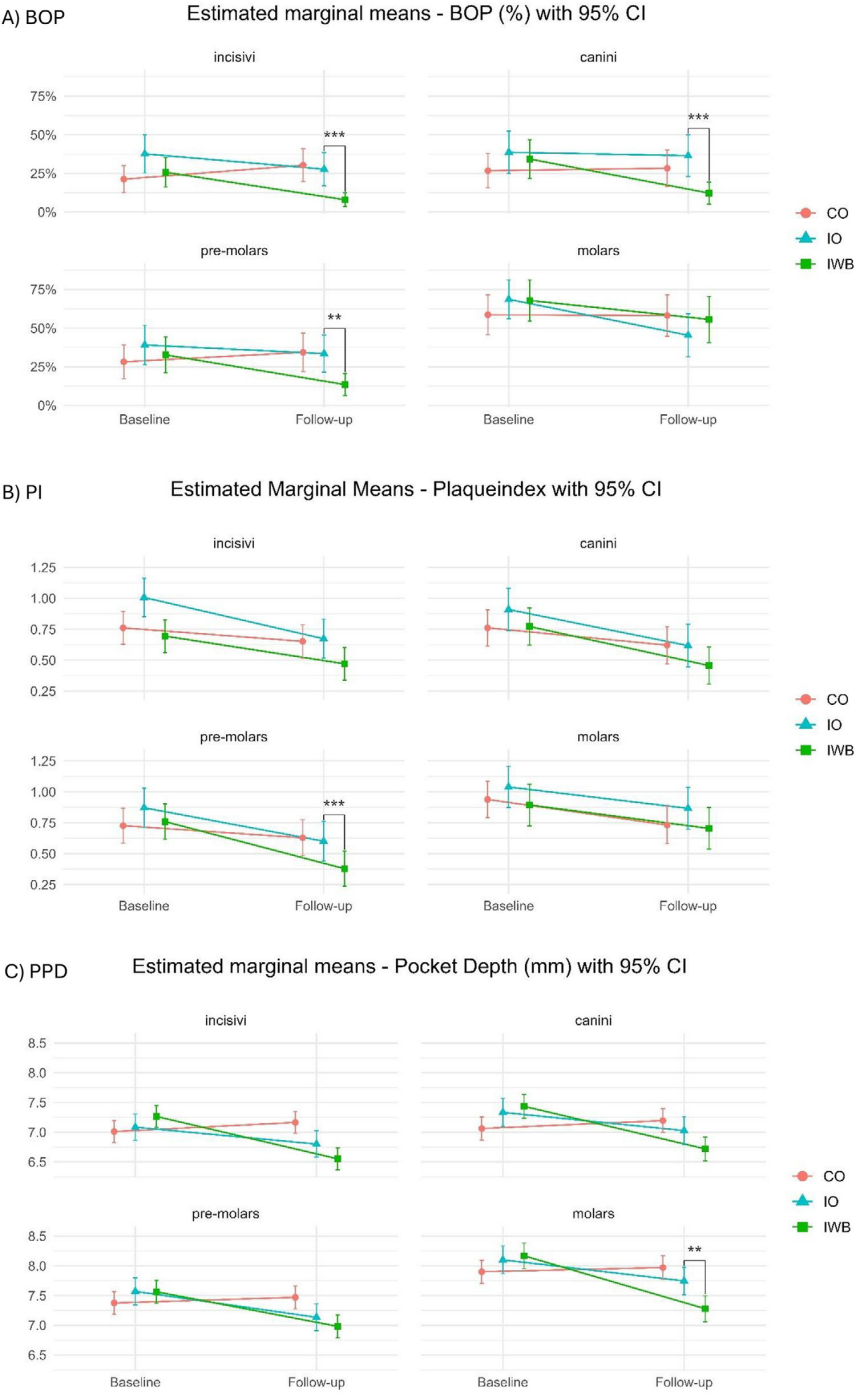
Estimated marginal means with 95% confidence intervals for A) BOP, B) PI, and C) PPD according to tooth group and study group (IWB, IO, and control) from baseline to the 3‐month follow‐up. Brackets indicate statistically significant intergroup differences between IWB and IO. **p ≤ 0.01; ***p < 0.001. (A) Bop. (B) Pi. (C) Ppd. BOP, bleeding on probing; CI, confidence interval; IO, intervention only; IWB, intervention with brushing; PI, plaque index; PPD, probing pocket depth.

**BOP**. Tooth‐type‐specific analysis showed greater reductions in BOP with IWB than with IO for incisors, canines, and premolars, whereas no significant intergroup difference was observed for molars (*p* = 0.4). For incisors, BOP decreased by 17% with IWB (*p* < 0.001 vs. baseline) and by 10% with IO (*p* = 0.08 vs. baseline), with a significant difference between the IWB and IO groups (*p* < 0.001). For canines, BOP decreased by 22% with IWB (*p* < 0.001) and by 3% with IO (*p* = 0.8), with a significant difference between groups (*p* < 0.001). For premolars, BOP decreased by 19% with IWB (*p* < 0.001) and by 5% with IO (*p* = 0.4), with a significant difference between groups (*p* = 0.01).

**PI**. A significant difference between the IWB and IO groups was observed only for premolars. In premolars, PI decreased by 0.38 with IWB (*p* < 0.001 vs. baseline) and by 0.27 with IO (*p* < 0.001 vs. baseline), with a significant difference between groups (*p* < 0.001).

**PPD**. A significant difference between the IWB and IO groups was observed only for molars. In molars, PPD decreased by 0.9 mm with IWB (*p* < 0.001 vs. baseline) and by 0.4 mm with IO (*p* < 0.001 vs. baseline), with a significant difference between groups (*p* = 0.01).

## 4 Discussion

In this prospective extension study with historical comparisons, we evaluated whether adding professional toothbrushing every two weeks to shortened outreach periodontal therapy improved clinical outcomes in nursing home residents [22].

As anticipated, baseline oral health was poor, underscoring the persistent challenges associated with maintaining oral hygiene and delivering periodontal care in nursing home populations [5]. IWB significantly improved all clinical measures, including a shift from deeper to shallower, healthier periodontal pockets, whereas IO only improved PI and PPD. Although not all comparisons between IWB and IO reached statistical significance, all investigated clinical parameters showed numerically greater improvements in the IWB group. These findings suggest that the addition of professional toothbrushing provided further clinical benefits beyond shortened outreach periodontal therapy alone, particularly with regard to periodontal inflammation.

Thus, professional brushing provides added clinical value but cannot sufficiently compensate for a lack of consistent, *daily* oral hygiene in institutional care. Instead of relying solely on professional interventions, daily oral hygiene in care facilities should be strengthened alongside regular therapy—this requires engaging caregivers, nursing staff, and residents alike. Despite the challenges of outreach care (limited infrastructure, time constraints, communication barriers with cognitively impaired residents, coordination with nursing staff, and infection control difficulties [31, 32], our results show that short, targeted interventions can significantly improve oral health in this vulnerable group. Future studies should explore strategies to further optimize professional oral hygiene support in nursing home residents.

From a clinical perspective, the observed improvements in periodontal health parameters are meaningful. The increase in healthy PPD sites and the reduction in the number of deep pockets were most pronounced in the IWB group, indicating the benefits of adjunctive professional brushing. Although PPD reductions were modest versus younger populations, treatment goals differ in geriatric, care‐dependent patients. Decreasing inflammatory surface area, thereby reducing systemic exposure to bacteria and inflammatory markers, may help increase the number of healthy pockets and improve systemic health [33, 34] and should be a focus of future research.

The lack of statistically significant differences in plaque reduction between IWB and IO likely reflects initial oral hygiene optimization in both groups, improving hygiene before therapy and limiting measurable gains. Notably, BOP was high at baseline across all three groups, indicating a substantial pre‐existing inflammatory burden. At the 3‐month examination, PI was again relatively low in the control and IO groups, whereas BOP remained high, suggesting that plaque levels observed at a single examination may not fully reflect oral hygiene over the preceding period. Although visits to the nursing facilities were announced in advance, this was routine practice for both study and non‐study visits by the cooperating dentist, and nursing staff were not necessarily aware whether a scheduled visit was study‐related. Thus, while short‐term changes in oral hygiene before an examination cannot be entirely excluded, systematic preparation specifically for study visits appears unlikely. Previous studies show that professional brushing is particularly effective in populations with severe oral hygiene deficits, suggesting its impact may be greater when no concurrent periodontal therapy is provided [18, 19]. The Hawthorne effect may also have improved oral hygiene in all groups [35]. Moreover, the PI used in this study does not distinguish between new, less pathogenic biofilm and mature, disease‐associated plaque; unassessed plaque and calculus formation factors might have influenced results [36]. Future studies should incorporate microbiological assessments to determine whether interventions affect plaque pathogenicity as well as quantity. Beyond reducing visible plaque accumulation, professional supragingival plaque control may also influence the composition and maturation of the subgingival biofilm. Ecological interactions between supragingival and subgingival biofilms are well established, and reductions in supragingival plaque may alter nutrient supply, bacterial succession, and recolonization patterns within periodontal pockets. This mechanism may partly explain why the IWB group demonstrated greater improvements in periodontal inflammation and probing depths despite the absence of additional subgingival instrumentation beyond the initial periodontal therapy.

Our findings are consistent with previous studies demonstrating beneficial effects of professional oral hygiene interventions in nursing home residents and other care‐dependent older adults. Sjögren et al. [37, 38] reported that domiciliary professional oral care provided in nursing homes resulted in significant improvements in oral hygiene and gingival health compared with usual care. Similarly, Croonquist and colleagues observed improvements in oral hygiene following regular professional oral care combined with individualized oral hygiene instructions in care‐dependent elderly individuals. Collectively, these studies support the concept that professional oral hygiene measures can improve oral health outcomes even in highly dependent populations where adequate self‐performed oral hygiene is often difficult to achieve. The frequency of professional oral hygiene interventions may also influence treatment outcomes. In addition to the findings reported by Londero et al. [39], who observed significant reductions in gingival inflammation following weekly professional plaque control combined with daily personal oral hygiene measures, studies evaluating professional oral health programs in nursing homes have suggested that more frequent interventions may yield greater improvements than less intensive schedules. In the present study, professional brushing was performed every two weeks and resulted in additional reductions in periodontal inflammation compared with periodontal therapy alone. Nevertheless, it is conceivable that weekly professional brushing may have produced even greater clinical benefits. Our findings therefore support previous observations indicating that intervention frequency is an important determinant of treatment success and should be further investigated in future studies involving nursing home residents receiving periodontal therapy. Marginal means analysis revealed significantly greater site‐specific improvements in clinical parameters with IWB versus IO. The most pronounced BOP reductions were in anterior teeth and premolars (but not molars), significant PI reductions were limited to premolars, and PPD improvements were significantly greater in molars only. These results suggest that the benefits of professional brushing vary by tooth type and location—likely reflecting accessibility, oral hygiene challenges, and anatomical differences. This highlights the need to tailor preventive care in long‐term care facilities to tooth‐specific characteristics and high‐risk regions. Our findings illustrate the complexity of thorough oral care by non‐dental professionals, and the need for comprehensive, targeted staff training to ensure consistent daily oral health protocols and thorough cleaning across all tooth surfaces, particularly in posterior regions.

Beyond local effects, improved oral hygiene has been linked to systemic health benefits. Structured oral care programs in long‐term care facilities have additionally been associated with improved oral care quality and reduced pneumonia risk, highlighting the broader relevance of oral hygiene interventions beyond periodontal outcomes alone [11, 40]. For example, poor oral health in institutionalized elderly patients increased the risk of aspiration pneumonia, cardiovascular diseases, and systemic inflammation, and has been defined as a new geriatric syndrome [41]. Studies are vital to explore whether brushing interventions combined with periodontal therapy can reduce systemic inflammation and improve overall health in this vulnerable population.

Our findings emphasize the necessity of structured, systematic periodontal care in outreach settings. Improvements in both intervention groups—and additional clinical benefits with professional brushing—reinforce the importance of integrating periodontal therapy *and* daily oral hygiene optimization into routine geriatric care. However, professional interventions cannot replace well‐informed daily oral hygiene by patients, caregivers, or healthcare providers. This should remain the goal of interdisciplinary care.

A strength of our study is the controlled design based on a previously randomized trial and the evaluation of a rarely investigated outreach periodontal treatment pathway in nursing home residents. However, due to the high dropout (13 patients) from the original control group, additional participants were recruited to the IWB group. Five participants from the original control group also joined the IWB group—and were therefore not randomized but assigned to IWB. Accordingly, the present study should not be interpreted as a randomized controlled trial. Rather, it represents a prospective extension study with historical comparisons. Residual confounding and selection bias cannot be excluded despite the comparable baseline characteristics observed between groups. Both actions represent a methodical bias in terms of the initial randomization and counting participants twice. Nevertheless, the absence of significant baseline or clinical endpoint differences (apart from a higher PI with IO versus IWB) suggests a balanced population, minimizing these limitations. Another limitation is that one dentist performed all periodontal therapy. However, the dentist was a calibrated specialist in geriatric dentistry, ensuring consistency and adherence to high clinical standards. Another limitation is the lack of documented examiner blinding. In particular, blinding with respect to the prospectively enrolled IWB group was not feasible because the additional professional oral hygiene intervention was apparent during study conduct; therefore, potential measurement bias cannot be entirely excluded.

## 5 Conclusion

Adding professional toothbrushing every two weeks to shortened outreach periodontal therapy consistently resulted in greater improvements across all clinical parameters compared with periodontal therapy alone, although not all differences reached statistical significance. This supports implementing additional oral hygiene interventions alongside periodontal treatment, which alone provided significant benefits over no intervention. Yet given the high prevalence of periodontal disease in this population and its systemic impact, optimizing daily oral hygiene practices remains the cornerstone of oral health in care‐dependent individuals. Future studies should refine intervention protocols by involving care staff, family members, and residents, and investigate long‐term and systemic health effects to further advance periodontal care in this vulnerable population.

## Author Contributions

All authors have made substantial contributions to conception and design of the study. AGB, FL, DB, MH and SHMD have been involved in data collection and data analysis. Anna G. Barbe, Dirk Bleiel, Max von Kohout, Martin Hellmich and Sonja H. M. Derman have been involved in data interpretation. Anna G. Barbe, Martin Hellmich, Dirk Bleiel and Sonja H. M. Derman have been involved in drafting the manuscript and revising it critically and have given final approval of the version to be published. All authors agree to be accountable for all aspects of the work in ensuring that questions related to the accuracy or integrity of any part of the work are appropriately investigated and resolved.

## Ethics Statement

The study received ethical approval from the local ethics review board of the University of Cologne, Cologne, Germany (22‐1219). All procedures complied with the ethical standards of the institutional research committee, the 1964 Helsinki Declaration, and subsequent amendments or comparable ethical standards. Informed consent was obtained from all participants or their legal guardians.

## Conflicts of Interest

The authors declare no conflicts of interest.

## Data Availability

The data that support the findings of this study are available from the corresponding author upon reasonable request.
